# Supplementary material for: Isotopic Niche of Syntopic Granivores in Commercial Orchards and Meadows
Source: Animals (Basel). 2021 Aug 11;11(8):2375. doi: 10.3390/ani11082375 (PMC8388717; doi:10.3390/ani11082375)
Supplement: Supplementary file 1 [file animals-11-02375-s001.zip › animals-1288701-supplementary.pdf]

animals

MDPI

Supplementary Material

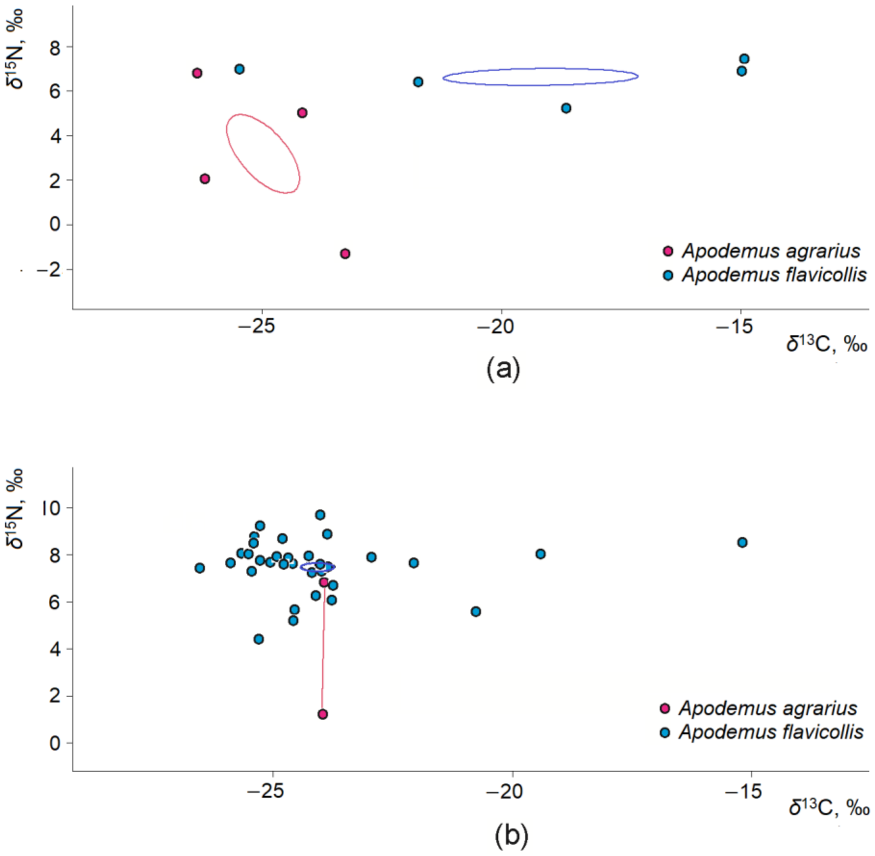

Figure S1. Central ellipses of two syntopic *Apodemus* species in the isotopic space, representing fundamental niches in plum orchards (**a**) and currant plantations (**b**).

Table S1. Central position (mean ± SE) and ranges of stable isotope ratios in the hair of *Apodemus flavicollis*, *Apodemus agrarius* and *Micromys minutus* in the habitats of commercial orchards, berry plantations and control meadows in Lithuania, 2018–2020. Abbreviations: AO, apple orchards; PO, plum orchards; RP, raspberry plantations; CP, currant plantations; CH, control habitats; Af, *A. flavicollis*; Aa, *A. agrarius*; Mm, *M. minutus*; hyphen, species not present.

Habitat	Species	δ^{13}C Values, ‰			δ^{15}N Values, ‰		
		Mean ± SE	Min–max	Range	Mean ± SE	Min–max	Range
AO	Af	−24.37 ± 0.13	−27.11–−16.97	10.14	5.09 ± 0.14	2.27–9.71	7.44
	Aa	−22.92 ± 0.57	−29.69–−12.28	17.41	6.75 ± 0.18	3.36–9.16	5.81
	Mm	−25.47 ± 0.17	−25.92–−24.91	1.01	10.13 ± 3.09	3.32–20.94	17.62
PO	Af	−24.97 ± 0.76	−26.34–−23.25	3.09	3.18 ± 1.76	−1.28–6.80	8.08
	Aa	−19.18 ± 2.03	−25.50–−14.94	10.56	6.63 ± 0.38	5.22–7.32	2.10
	Mm	-	-	-	-	-	-
RP	Af	−24.87 ± 0.17	−26.69–−23.40	3.29	3.23 ± 0.56	−1.74–6.83	8.57
	Aa	−22.85 ± 0.45	−26.52–−15.04	11.48	6.04 ± 0.44	3.02–15.52	12.50
	Mm	−17.41	-	-	6.00	-	-
CP	Af	−23.95 ± 0.03	−23.98–−23.92	0.06	4.05 ± 2.75	1.30–6.80	5.50
	Aa	−24.06 ± 0.35	−26.58–−15.16	11.42	7.48 ± 0.18	4.44–9.22	4.78
	Mm	-	-	-	-	-	-

	Af	−23.94 ± 0.31	−26.86–−13.65	13.21	5.81 ± 0.17	2.91–8.87	5.96
CH	Aa	−23.55 ± 0.25	−27.20–−13.34	13.86	6.84 ± 0.17	2.52–11.11	8.59
	Mm	−23.35 ± 1.79	−27.49–−16.68	10.81	8.74 ± 0.76	6.95–11.46	4.51

Table S2. Size of isotopic niches of granivorous mice species in agricultural and control habitats: total area (TA), standard ellipse area (SEA) and central ellipses, corrected fort sample size (SEAc); hyphen, number of processed samples < 5 or species not present.

Habitat	*Apodemus flavicollis*			*Apodemus agrarius*			*Micromys minutus*		
	**TA**	**SEA**	**SEAc**	**TA**	**SEA**	**SEAc**	**TA**	**SEA**	**SEAc**
Apple orchards	55.43	7.30	7.35	56.31	13.77	14.09	6.70	6.27	8.36
Plum orchards	12.40	13.14	19.71	11.04	12.06	16.08	-	-	-
Raspberry plantations	11.04	12.06	16.08	12.40	13.14	19.71	-	-	-
Currant plantations	-	-	-	30.80	6.79	7.00	-	-	-
Control habitats	45.72	11.68	11.82	77.60	13.77	13.89	20.18	19.79	26.39
